# Crystal Structure and Hydrogen Bonding Study of (10*E*)-2,2-Dimethyl-3,4-dihydro-2*H*-benzo[*g*]chromene-5,10-dione 10-Oxime Derived From α-Lapachone

**DOI:** 10.3390/molecules16021192

**Published:** 2011-01-27

**Authors:** Andrea R. da Silva, Marcelo H. Herbst, Aurelio B. B. Ferreira, Ari M. da Silva, Lorenzo C. Visentin

**Affiliations:** 1 Departamento de Química, Universidade Federal Rural do Rio de Janeiro, BR 465, km 47, Seropédica, RJ, 23890-000, Brazil; 2 Instituto de Química, Universidade Federal do Rio de Janeiro, Avenida Athos da Silveira Ramos, 149, Bloco A, 7° andar, Cidade Universitária, Rio de Janeiro, RJ, 21941-909, Brazil

**Keywords:** oxime, crystal structure, hydrogen bond

## Abstract

The compound (10*E*)-2,2-dimethyl-3,4-dihydro-2H-benzo[g]chromene-5,10-dione-10-oxime (**1**) was synthesized from α-lapachone and hydroxylamine chloride in alkaline medium. Single-crystals suitable for X-ray diffraction measurements were grown from an ethanol solution, and the crystal structure of the title molecule is reported for the first time. The title molecule was also characterized by ^1^H- and ^13^C-NMR in CDCl_3_ solution, FTIR and MS. The crystal structure of **1** shows an *E* stereochemistry and dimers formed through classical hydrogen bonds.

## 1. Introduction

The chemistry of oximes derived from lapachol and lapachones has been studied by several groups because these substances show important biologic and organic chemical applications [1,2,3,4]. α-Lapachone (2,2-dimethyl-3,4-dihydro-2*H*-benzo[*g*]chromene-5,10-dione) is a natural naphtha-quinone found in the wood of trees of the genus *Tabebuia* (family *Bignoniaceae*), which occur in most of Central and South Americas and are known as *ipê* or *pau d’arco* in Brazil (e.g., ipê-roxo, *Tabebuia serratifolia*) and *lapacho* in Argentina and other Spanish-speaking countries. α-Lapachone, (2,2-dimethyl-3,4-dihydro-2*H*-benzo[*g*]chromene-5,10-dione) and *β*-lapachone, (2,2-dimethyl-3,4-dihydro-2*H*-benzo[*h*]chromene-5,6-dione) occur in small quantities, while the isomer lapachol (2-hydroxy-3-(3-methyl-2-butenyl)-1,4-naphtoquinone) is the most abundant naphtoquinone found in the *ipê-roxo* wood [5]; these substances have been under medical study for their varied biological activities and *ipê-roxo* has been traditionally used in folk medicine [6,7,8]. The lapachones may be easily synthesized, from lapachol (2-hydroxy-3-(3-methyl-2-butenyl)-1,4-naphtoquinone) through cyclization in acidic media [7]. Lemos *et al*., studying Brazilian natural quinones and their derivatives by ^1^H- and ^13^C-NMR spectroscopy in CDCl_3_ solutions (including 2D experiments ^1^H-^1^H-COSY, HMQC ^1^J_CH_ and HMBC ^n^J_CH_ (n = 2 and 3)), have assigned *Z* stereochemistry for the 10-oxime derived from α-lapachone [4]. Herein we report a single crystal X-ray diffraction study that presents the first report of the crystal structure of the (10*E*)-2,2-dimethyl-3,4-dihydro-2H-benzo[g]chromene-5,10-dione10-oxime (**1**).

## 2. Results and Discussion

**Figure 1:**
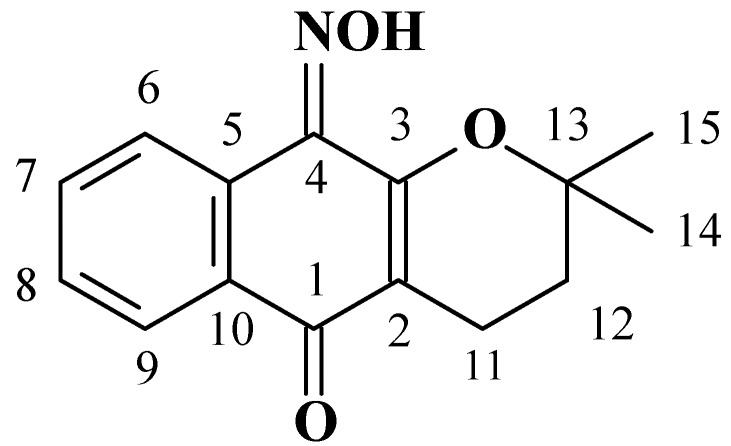
Structural scheme and numbering label for **1**.

The oxime **1**, one of two possible regioisomers obtainable from α-lapachone, adopts an *E* stereochemistry in the crystalline state. The conformation is governed by intra- and intermolecular interactions of classical and non classical hydrogen bond types. The structural formula of the title molecule is depicted in Figure 1. Although the numbering scheme recommended by IUPAC has been used for the name of the title molecule, in the NMR assignments a different numbering scheme was chosen, as shown in the figure. This numbering scheme was further used for refining the X-ray structure.

### 2.1. Infrared, ^1^H- and ^13^C-Nuclear Magnetic Resonance Spectroscopy

The infrared spectra obtained in the solid state (KBr) for **1** in the range from 4,000 to 400 cm^−1^ shows characteristic oxime group absorption bands. The principal absorption frequencies in this molecule are attributed to (O-H), (C=O), (C=N), (C-H) and (C=C) bonds, which show absorption bands in the range of the 3,160 cm^−1^ (νOH), 2,975–2,937 cm^−1^ (CH_2_ and CH_3_), 1,639 cm^−1^ (C=N), 1,621 cm^−1^ (C=O) and 1,272 cm^−1^ (C-O). These absorption frequencies supply important information on the molecular structure of **1**. The IR spectrum of **1** shows broad hydroxyl absorption due to hydrogen bonding around this group centered in 3,160 cm^−1^. These data agree with literature reports [1]. The X-ray diffraction reveals the same hydrogen bonds around the hydroxyl group. 

The ^1^H-NMR spectrum of the 10-oxime from α-lapachone (of which **1** is the *E* stereoisomer) in CDCl_3_ solution shows two triplet signals at δ 2.63 and δ 1.88 ppm assigned to the two methylene groups at the C11 and C12 labeled atoms, which are part of the 2-oxene ring moiety. Also observed is a cumulative singlet signal at δ 1.48 ppm, assigned to the methyl groups (C14 and C15) in this same ring. The singlet signal attributed to the OH group was observed at δ 12.25 ppm in CDCl_3_, in accordance with literature [4]. In DMSO-*d_6_* this signal shifted to 13.65 and in pyridine-*d5* to 15.85 ppm, but there were no significant alterations in the other peaks. A NOESY experiment in CDCl_3_ solution (not shown) was inconclusive and did not elucidate the chemical neighborhood of the OH hydrogen atom. The ^13^C-NMR spectrum indicated the presence of fourteen carbon atom signals. A cumulative signal at δ 26.8 ppm is assigned to the carbon atoms labeled C14 and C15, and there is only one signal indicating the presence of a carbonyl group in δ 184.0 ppm. The signal in 139.7 ppm was assigned to the quaternary carbon of the oxime group. Table 1 lists all the ^1^H- and ^13^C-NMR data.

**Table 1:** ^1^H- and ^13^C-NMR data for 1.

Carbon Atom	δ ^13^C	δ ^1^H ( *J* in Hz )
1	184.0	-
2	113.6	-
3	156.6	-
4	139.7	-
5	126.9	-
6	129.8	9.06 (d, 7.8)
7	132.6	7.65 (td, 1.4, 7.6)
8	130.7	7,58 (td, 1.2, 7.7)
9	126.8	8.27 (dd, 1.2, 7.6)
10	130.7	-
11	16.9	2.63 (t, 6.6)
12	31.8	1.88 (t, 6.6)
13	78.4	-
14	26.8	1.48 (s)
15	26.8	1.48 (s)
16	-	12.25 (s)

### 2.2. Crystal Structure

**Figure 2:**
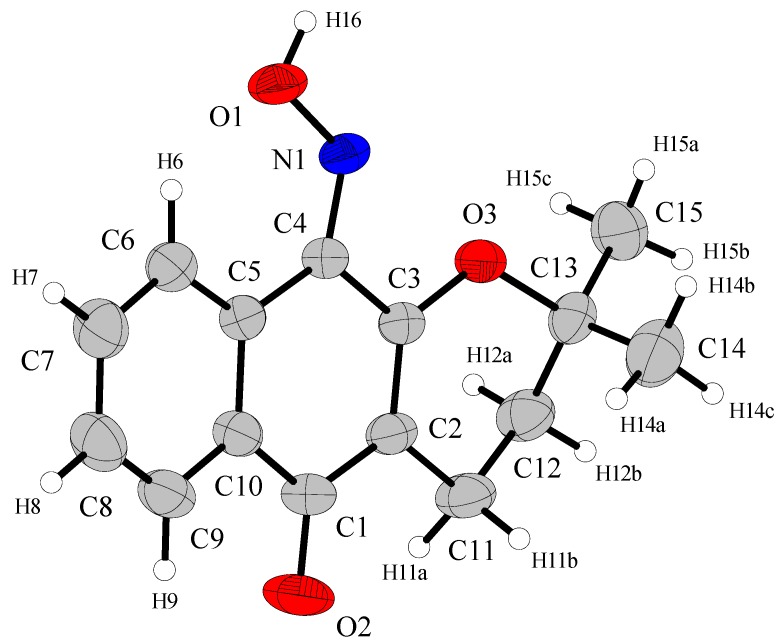
View of the *ORTEP* projection for (**1**) with respective atom-numbering scheme [19]. Displacement ellipsoids are draw at the 50% probability level.

The crystal structure of **1** is reported here for the first time. The atomic arrangement and numbering scheme for **1** are shown in Figure 2. Selected bond lengths and bond angles are listed in Table 2. These parameters are in the expected ranges reported in the literature [1,9,10,11] for compounds of this class. For instance, the oxime 6-hydroxy-3-(hydroxyimino)indolin-2-one [9], shows bond lengths around the N-OH group of 1.361(3)Å for N2-O3 and 1.286(4)Å for N2=C8. For 4-(1-methylvinyl)cyclohexene-1-carbaldehyde oxime [10] these distances are of 1.407(3)Å for O1-N1 and 1.266(4)Å for N1=C1, and the (*E*)-4-nitrobenzaldehyde oxime [11] shows similar distances around the oxime group, which are 1.401(3)Å for O3-N2 and 1.264(3)Å for N2-C7. 

**Table 2:** Selected geometric parameters in **(1)_,_** (Å/°).

Bonds	
N1-O1	1.382(1)
N1-C4	1.290(2)
C1-O2	1.234(2)
C3-O3	1.353(2)
C13-O3	1.473(2)
Angles	
O1-N1-C4	117.2(1)
C3-O3-C13	118.1(1)
O2-C1-C10	120.8(1)
O2-C1-C2	120.6(1)
C12-C13-C15	111.8(1)
O3-C13-C14	107.8(1)

The average bond lengths in **1** also agree with the typical interatomic distance for C=N-OH oxime groups (R = aryl) in oxime molecules. The *International Tables for X-ray Crystallography* [12] list these parameters for typical bond distances, which are 1.281 Å for C*sp^2^*=N [σ = 0.013, *q_l _*= 1.273 Å and *q_u_* = 1.288 Å] and 1.416 Å for N-OH [σ = 0.006, *q_l _*= 1.416 Å and *q_u_* = 1.420 Å].

**Figure 3:**
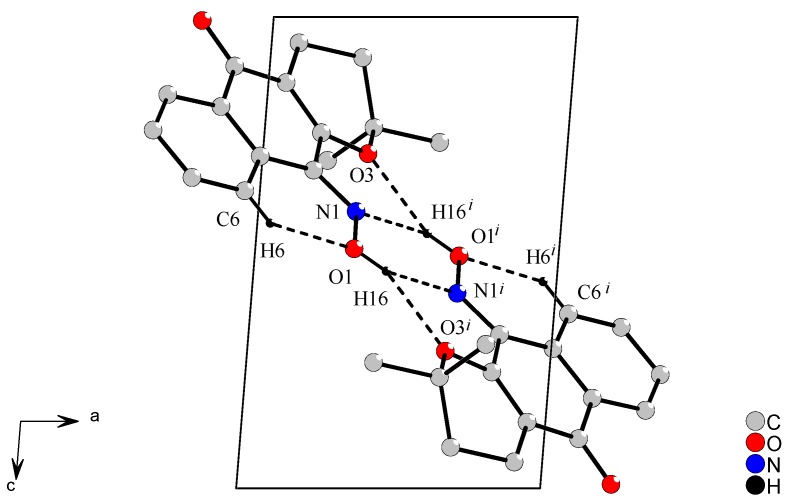
View of the centrosymmetric *R*_2_^2^(6) dimers in (101) plane by intra- and intermolecular interactions. [Symmetry code (*i*) = 1-*x*, 2-*y*, 1-*z*]

The phenyl ring and the ring fused to it in **1** form an almost planar system: the dihedral angle between C1-C10 [r.m.s. 0.0306 Å] and C10-C9 [r.m.s. 0.0073Å] rings is 4.7(1)°. The six-membered (oxene) ether ring is distorted and the torsion angle in O3-C3-C2-C11 is −3.6(2)°, whereas in O3-C13-C12-C11 it is −59.1 (2)°. In **1**, the C6-H6…O1 intramolecular hydrogen bond gives rigidity to the oxime group by the formation of one six-membered ring (Figure 3).

In addition, a dimer arrangement is created in **1 **by O1-H16…O3*^i^* and O1-H16℘?℘N1*^i^* bifurcate hydrogen bonds, which link the molecules into centrosymmetric *R*_2_^2^(6) dimers on the (101) plane, symmetry code (*i*) = 1-*x*, 2-*y*, 1-*z*. These bifurcate interactions cement the crystal structure by formation of one six- and two five-membered rings, which are formed by 1-*x*, 2-*y*, 1-*z* equivalent symmetry. All noncovalent bonds, that is, classical and non-classical hydrogen bonds, enforce the *E* stereochemistry around oxime group in this crystal structure. These hydrogen bonds were calculated by *PLATON* program [13] and their parameters are in accordance with the literature [14].

**Figure 4:**
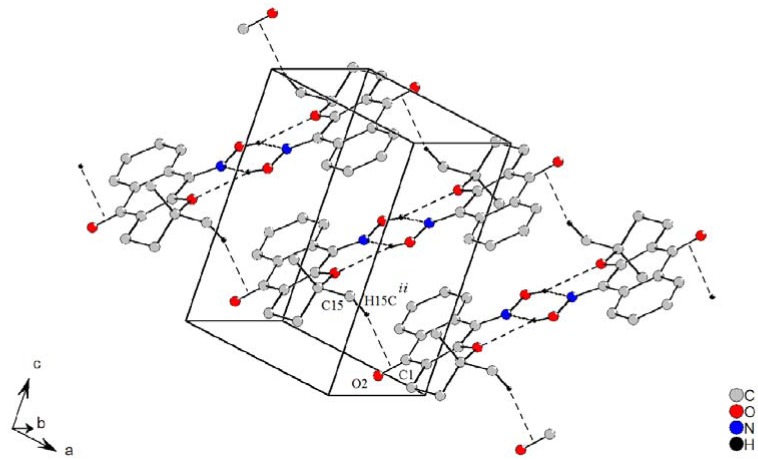
Self assembly by *tectons* linked through C-H^…^π noncovalent interactions. The intramolecular interaction is omitted. [Symmetry code (*ii*) = 1*+x, y, z*]

Besides that, the centrosymmetric dimers are self-arranged in a 1-D fashion through four C-H^…^π intermolecular interactions along the [100] crystallography direction (Figure 4). The π electrons from the carbonyl group are responsible for these interactions and the molecular self-organization.

The self assembly of predictable supramolecular aggregates of this self-organization can be induced by selective, directional attractive noncovalent interactions [15]. The molecules which play the role of building blocks in a self-assembled, ordered supramolecular structure are called *tectons* [16,17,18]. The crystal packing is accomplished by these weak interactions, and its unusual noncovalent bond grows a staircase on (101) plane, where the steps are formed with *tectons* linked by four C15-H15c^…^*Cg^ii^* from the center double bond in the C1=O2 group [symmetry code (*ii*) = *1 + x, y, z*] formed by side-on noncovalent interactions (Figure 4). The bond length C15-H15c is slightly longer than a normal C-H bond in methyl groups. These noncovalent bonds are equivalent by symmetry, with the following symmetry codes, 1*-x,* 2*-y,* 1*-z; -x,* 2*-y,* 1*-z;* and*-*1*+x, y, z*. Geometric parameters for H-bonds in (**1**) are show in Table 3.

**Table 3:** Geometric parameters for H-bonds in (**1**), (Å/°).

*D*-H···A	*D*-H	H··· *A*	*D*···A	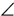 *D*-H···A
O1-H16^...^O3 *^i^*	0.86(2)	2.35(2)	3.070(2)	142(2)
O1-H16^...^N1 *^i^*	0.86(2)	2.01(2)	2.771(2)	149(2)
C6-H6^…^O1	0.93	2.16	2.785(2)	123
*D*-H···*Cg*	*D*-H	H··· *Cg*	*D*···*Cg*	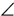 *D*-H···*Cg*
C15-H15c^…^ *Cg^ii^*	1.02(2)	2.795	3.797	160.28

[Symmetry code (*i*) = 1-*x*, 2-*y*, 1-*z* and(*ii*) = 1*+x, y, z*; and *Cg* = center CO double bond]

## 3. Experimental

### 3.1. General

The ^1^H- and ^13^C-NMR spectra were obtained in a Bruker Avance 400 (400 MHz ^1^H and 100 MHz ^13^C), in CDCl_3_, DMSO-*d_6_* and pyridine-*d_5_*_, _using TMS as internal standard. The IR spectra were measured in a Perkin-Elmer 1605 spectrophotometer, using KBr pellets. Mass spectra were measured in a Varian Saturn 2000 spectrometer. The melting point was determined with a Büchi 510 apparatus. 

### 3.2. Synthesis of ***1***

The title molecule was synthesized as follows [4] (all the manipulations were carried out at room temperature): α-lapachone (2,2-dimethyl-3,4-dihydro-2*H*-benzo[*g*]chromene-5,10-dione) (0.242 g, 1 mmol) was added to a methanol solution (10 mL) of hydroxylamine chloride (NH_2_OHHCl, 0.075 g, 1 mmol) in the presence of sodium hydroxide (5%, w/v). After 2 h of magnetic stirring, the solution was neutralized with acetic acid and filtered. Anhydrous sodium sulphate was added to the solution, which was filtered once more. The solvent was removed under vacuum, and the crude yellow product was isolated. Yellow block-shaped crystals suitable for X-ray diffraction were obtained by recrystallization from ethanol. Yield: 0.962 g, 37%; Melting point = 172.4 °C; MS (EI): *m/z* (%) 257 (90), 241 (45), 240 (50), 226 (100), 212 (10), 201 (25), 186 (10), 158 (10), 143 (5), 130 (50), 115 (10), 102 (40), 89 (5), 76 (25), 63 (10), 50 (20). NMR data: see Table 1.

### 3.3. X-ray diffraction Experiment

The X-ray data for the title compound were collected from a *Bruker KAPPA* CCD *diffractometer* [20], at 295K and *MoKα* monochromatic-graphite radiation. The crystal data are listed in Table 4. The cell parameters for the oxime molecule were obtained using the *PHICHI* and *DIRAX* programs [21,22]. The average data were reduced using the *EvalCCD* program and the absorption correction was performed with the *SADABS* programs [23,24]. The structure was solved by direct methods via *SHELXS97* and refined via *SHELXL97* by a full-matrix least-squares treatment with anisotropic temperature parameters for all non H atoms [25]. H atoms of the unsaturated carbon were positioned geometrically (C–H = 0.93 Å for Csp^2^ atoms) and treated as riding on their respective C atoms, with *U_iso_*(H) values set at 1.2*U*_eq_Csp^2^. The hydrogen atoms of the oxime group, methyl, and methylene groups were located in *Fourier map* and free refined to position. 

**Table 4:** Crystal data and structure refinement parameters for (**1**).

Empirical formula	C_15_H_15_NO_3_
Formula weight	257.28
Temperature	295(2) K
Wavelength	0.71073 A
Crystal system, space group	*triclinic*, *P*-1
Unit cell dimensions	*a* = 6.6069(13) Å *α* = 91.47(3)°
	*b* = 9.6001(19) Å *β* = 94.47(3)°
	*c* = 10.176(2) Å *γ* = 94.27(3)°
Volume	641.3(2) Å^3^
*Z*, Calculated density	2, 1.332 mg/m^3^
Absorption coefficient	0.093 mm^-1^
*F*(000)	272
Crystal size	0.47 × 0.40 × 0.20 mm
Theta range for data collection	2.97 to 25.00°
Limiting indices	−7<=h<=7, −11<=k<=11, −12<=l<=12
Reflections collected / unique	10469 / 2246 [*R_(int) _*= 0.0339]
Completeness to theta = 25.00	99.5 %
Max. and min. transmission	0.9816 and 0.9574
Refinement method	Full-matrix least-squares on *F*^2^
Data / restraints / parameters	2246 / 0 / 216
Goodness-of-fit on *F*^2^	1.032
Final R indices [*I*>2sigma(*I*)]	*R*_1_ = 0.0390, *wR*_2_ = 0.1010
*R* indices (all data)	*R*_1_ = 0.0550, *wR*_2_ = 0.1103
Largest diff. peak and hole	0.137 and −0.190 e.Å^-3^

## 4. Conclusions

The single-crystal X-ray diffraction studies reported in this work have established an *E* stereochemistry for compound **1**, whereas the literature reports a *Z* stereochemistry for **1** in solution, as derived from NMR spectroscopy data. In this way, either **1 **presents different stereochemistries in the solid state and in solution, or the assignment of the stereochemistry of **1** in solution is actually wrong. However, to our knowledge, there are no conclusive studies in the literature on the stereochemistry of complex oxime molecules in solution, including the 10-oxime of α-lapachone. In this work we have shown that in the solid state H-bonds link two oxime molecules, which form centrosymmetric dimers with an *R*_2_^2^(6) graph-set motif on the (101) plane. Furthermore, C-H^…^π noncovalent bonds grow in a 1D self-arrangement along the [100] crystallography direction. In the case of an eventual change in the stereochemistry of **1** upon crystallization, this should be related to the intra- and intermolecular hydrogen bonding. Further studies are in progress, as well as calculations, in an attempt to establish conclusively the stereochemistry of **1** in solution.

## Supplementary Material

CCDC 690106 contains the supplementary crystallographic data for this paper. These data can be obtained free of charge from The Cambridge Crystallographic Data Centre via www.ccdc.cam.ac.uk/data_request/cif.
