# Supplementary material for: Crystal Structure and Hydrogen Bonding Study of (10E)-2,2-Dimethyl-3,4-dihydro-2H-benzo[g]chromene-5,10-dione 10-Oxime Derived From α-Lapachone
Source: Molecules. 2011 Jan 27;16(2):1192–200. doi: 10.3390/molecules16021192 (PMC6259599; doi:10.3390/molecules16021192)
Supplement: Supplementary file 1 [file molecules-16-01192-s001.pdf]

```
#\#CIF_1.1

#  CIF produced by WinGX routine CIF_UPDATE
#  Created on 2008-06-02 at 20:19:41
#  Using CIFtbx version 2.6.2 16 Jun 1998

#  Dictionary name : cif_core.dic
#  Dictionary vers : 2.3
#  Request file    : c:\wingx\files\archive.dat
#  CIF files read  : sad


data_(10E)-2,2-dimethyl-3,4-dihydro-2H-benzo[g]chromene-5,10-dione-10-oxime

_audit_creation_date                    2008-06-02T20:19:41-00:00
_audit_creation_method                  'WinGX routine CIF_UPDATE'
_audit_conform_dict_name                cif_core.dic
_audit_conform_dict_version             2.3
_audit_conform_dict_location            ftp://ftp.iucr.org/pub/cif_core.dic
_publ_requested_category                FO

# Name and address of author for correspondence

_publ_contact_author_name      'Lorenzo do Canto Visentin'
_publ_contact_author_address
;
Instituto de Qu\'imica
Universidade Federal do Rio de Janeiro
21949-900 Rio de Janeiro-RJ, Brazil.
;
_publ_contact_author_email       'visentin72@yahoo.com.br'
_publ_contact_author_phone       '55 21 2562-7146'

_publ_requested_journal          'Molecules'
_publ_section_title
;
Crystal structure and Hydrogen bonds study in
(10E)-2,2-dimethyl-3,4-dihydro-2H-benzo[g]
chromene-5,10-dione 10-oxime, from alpha-lapachone
;
_publ_section_title_footnote
;
(10E)-2,2-dimethyl-3,4-dihydro-2H-benzo[g]chromene-5,10-dione 10-oxime
;
loop_
_publ_author_name
_publ_author_footnote
_publ_author_address
'Andrea Rosane da Silva' .
;
Departamento de Qu\'imica-ICE,
Universidade Federal Rural do Rio de Janeiro,
23890-000, Serop\'edica (RJ), Brazil.
;
'Marcelo Hawrylak Herbst' .
;
Departamento de Qu\'imica-ICE,
```

```
Universidade Federal Rural do Rio de Janeiro,
23890-000, Serop\'edica (RJ), Brazil.
;
'Aur\'elio Baird Buarque Ferreira' .
;
Departamento de Qu\'imica-ICE,
Universidade Federal Rural do Rio de Janeiro,
23890-000, Serop\'edica (RJ), Brazil.
;
'Ari M. da Silva' .
;
Departamento de Qu\'imica-ICE,
Universidade Federal Rural do Rio de Janeiro,
23890-000, Serop\'edica (RJ), Brazil.
;

'Lorenzo do Canto Visentin' .
;
Instituto de Qu\'imica
Universidade Federal do Rio de Janeiro
21949-900 Rio de Janeiro-RJ, Brazil.
;
_chemical_name_systematic
;
(10E)-2,2-dimethyl-3,4-dihydro-2H-benzo[g]chromene-5,10-dione 10-oxime
;
_chemical_melting_point            '446'
_chemical_formula_moiety           'C15 H15 N O3'
_chemical_formula_sum
 'C15 H15 N O3'
_chemical_formula_weight           257.28

loop_
 _atom_type_symbol
 _atom_type_description
 _atom_type_scat_dispersion_real
 _atom_type_scat_dispersion_imag
 _atom_type_scat_source
 'C'  'C'   0.0033   0.0016
 'International Tables Vol C Tables 4.2.6.8 and 6.1.1.4'
 'H'  'H'   0.0000   0.0000
 'International Tables Vol C Tables 4.2.6.8 and 6.1.1.4'
 'N'  'N'   0.0061   0.0033
 'International Tables Vol C Tables 4.2.6.8 and 6.1.1.4'
 'O'  'O'   0.0106   0.0060
 'International Tables Vol C Tables 4.2.6.8 and 6.1.1.4'

_symmetry_cell_setting            'triclinic'
_symmetry_space_group_name_H-M    'P-1'

loop_
 _symmetry_equiv_pos_as_xyz
 'x, y, z'
 '-x, -y, -z'

_cell_length_a                    6.6069(13)
_cell_length_b                    9.6001(19)
```

```
_cell_length_c                    10.176(2)
_cell_angle_alpha                 91.47(3)
_cell_angle_beta                  94.47(3)
_cell_angle_gamma                 94.27(3)
_cell_volume                      641.3(2)
_cell_formula_units_Z             2
_cell_measurement_temperature     295(2)
_cell_measurement_reflns_used     ?
_cell_measurement_theta_min       1.0
_cell_measurement_theta_max       27.5

_exptl_crystal_description        block
_exptl_crystal_colour             yellow
_exptl_crystal_size_max           0.47
_exptl_crystal_size_mid           0.40
_exptl_crystal_size_min           0.20
_exptl_crystal_density_meas       ?
_exptl_crystal_density_diffrn     1.332
_exptl_crystal_density_method     'not measured'
_exptl_crystal_F_000              272
_exptl_absorpt_coefficient_mu     0.093
_exptl_absorpt_correction_type    multi-scan
_exptl_absorpt_correction_T_min   0.9575
_exptl_absorpt_correction_T_max   0.9816
_exptl_absorpt_process_details    'SADABS (sheldrick,2004)'

_exptl_special_details
;
 ?
;

_diffrn_ambient_temperature       295(2)
_diffrn_radiation_wavelength      0.71073
_diffrn_radiation_type            MoK\a
_diffrn_radiation_source          'fine-focus sealed tube'
_diffrn_radiation_monochromator   graphite
_diffrn_measurement_device_type   'Enraf Nonius Kappa CCD'
_diffrn_measurement_device         '\k-geometry diffractometer'
_diffrn_measurement_method        '\f scans, and \w scans with \k'
_diffrn_detector_area_resol_mean  ?
_diffrn_standards_number          ?
_diffrn_standards_interval_count  ?
_diffrn_standards_interval_time   ?
_diffrn_standards_decay_%         ?
_diffrn_reflns_number             10469
_diffrn_reflns_av_R_equivalents   0.0339
_diffrn_reflns_av_sigmaI/netI     0.0245
_diffrn_reflns_limit_h_min        -7
_diffrn_reflns_limit_h_max        7
_diffrn_reflns_limit_k_min        -11
_diffrn_reflns_limit_k_max        11
_diffrn_reflns_limit_l_min        -12
_diffrn_reflns_limit_l_max        12
_diffrn_reflns_theta_min          2.97
_diffrn_reflns_theta_max          25.00
_reflns_number_total              2246
_reflns_number_gt                 1728
```

```
_reflns_threshold_expression      >2sigma(I)

_computing_data_collection        'COLLECT (Nonius, 1998)'
_computing_cell_refinement        'PHICHI (Duisenberg , 2000)'
_computing_data_reduction         'EvalCCD (Duisenberg, 2003)'
_computing_structure_solution     'SHELXS-97 (Sheldrick, 1997)'
_computing_structure_refinement   'SHELXL-97 (Sheldrick, 1997)'
_computing_molecular_graphics     'Ortep-3 for Windows (Farrugia, 1997)'
_computing_publication_material   'WinGX publication routines (Farrugia, 1999)'

_refine_special_details
;
 Refinement of F^2^ against ALL reflections.  The weighted R-factor wR and
 goodness of fit S are based on F^2^, conventional R-factors R are based
 on F, with F set to zero for negative F^2^. The threshold expression of
 F^2^ > 2sigma(F^2^) is used only for calculating R-factors(gt) etc. and is
 not relevant to the choice of reflections for refinement.  R-factors based
 on F^2^ are statistically about twice as large as those based on F, and R-
 factors based on ALL data will be even larger.
;

_refine_ls_structure_factor_coef  Fsqd
_refine_ls_matrix_type            full
_refine_ls_weighting_scheme       calc
_refine_ls_weighting_details
 'calc w=1/[\s^2^(Fo^2^)+(0.0630P)^2^+0.0606P] where P=(Fo^2^+2Fc^2^)/3'
_atom_sites_solution_primary      direct
_atom_sites_solution_secondary    difmap
_atom_sites_solution_hydrogens    geom
_refine_ls_hydrogen_treatment     mixed
_refine_ls_extinction_method      none
_refine_ls_extinction_coef        ?
_refine_ls_number_reflns          2246
_refine_ls_number_parameters      216
_refine_ls_number_restraints      0
_refine_ls_R_factor_all           0.0549
_refine_ls_R_factor_gt            0.0390
_refine_ls_wR_factor_ref          0.1087
_refine_ls_wR_factor_gt           0.0998
_refine_ls_goodness_of_fit_ref    1.049
_refine_ls_restrained_S_all       1.049
_refine_ls_shift/su_max           0.000
_refine_ls_shift/su_mean          0.000

loop_
 _atom_site_label
 _atom_site_type_symbol
 _atom_site_fract_x
 _atom_site_fract_y
 _atom_site_fract_z
 _atom_site_U_iso_or_equiv
 _atom_site_adp_type
 _atom_site_occupancy
 _atom_site_symmetry_multiplicity
 _atom_site_calc_flag
 _atom_site_refinement_flags
 _atom_site_disorder_assembly
```

```
 _atom_site_disorder_group
C12 C 0.3040(2) 0.63286(19) 0.08674(17) 0.0480(4) Uani 1 1 d . . .
C13 C 0.3592(2) 0.63187(15) 0.23498(16) 0.0438(4) Uani 1 1 d . . .
C15 C 0.5801(3) 0.6038(2) 0.2666(2) 0.0605(5) Uani 1 1 d . . .
C14 C 0.2153(4) 0.5331(2) 0.3049(2) 0.0645(5) Uani 1 1 d . . .
O1 O 0.32623(18) 1.11874(11) 0.49231(11) 0.0496(3) Uani 1 1 d . . .
N1 N 0.32296(18) 0.99931(12) 0.41313(12) 0.0392(3) Uani 1 1 d . . .
H11B H -0.013(3) 0.6032(19) 0.0740(17) 0.052(4) Uiso 1 1 d . . .
H12B H 0.317(3) 0.540(2) 0.0497(18) 0.060(5) Uiso 1 1 d . . .
H12A H 0.407(3) 0.6983(18) 0.0486(17) 0.055(5) Uiso 1 1 d . . .
H11A H 0.068(3) 0.7033(19) -0.037(2) 0.060(5) Uiso 1 1 d . . .
H1O H 0.437(4) 1.111(2) 0.540(2) 0.081(7) Uiso 1 1 d . . .
H15A H 0.615(3) 0.608(2) 0.356(3) 0.087(7) Uiso 1 1 d . . .
H15B H 0.604(3) 0.511(2) 0.2361(19) 0.071(6) Uiso 1 1 d . . .
H14A H 0.075(4) 0.566(2) 0.296(2) 0.083(7) Uiso 1 1 d . . .
H15C H 0.676(4) 0.675(3) 0.224(2) 0.099(8) Uiso 1 1 d . . .
H14B H 0.263(3) 0.532(2) 0.400(2) 0.089(7) Uiso 1 1 d . . .
H14C H 0.220(3) 0.441(2) 0.264(2) 0.084(7) Uiso 1 1 d . . .
O3 O 0.34672(15) 0.77394(10) 0.29046(10) 0.0452(3) Uani 1 1 d . . .
C1 C 0.1719(2) 0.97625(14) 0.32517(13) 0.0341(3) Uani 1 1 d . . .
C2 C 0.1881(2) 0.84616(14) 0.24632(14) 0.0351(3) Uani 1 1 d . . .
C3 C 0.0591(2) 0.80624(15) 0.13975(14) 0.0380(3) Uani 1 1 d . . .
C4 C -0.1146(2) 0.88588(16) 0.10497(14) 0.0409(4) Uani 1 1 d . . .
C10 C -0.0071(2) 1.05705(14) 0.29570(13) 0.0353(3) Uani 1 1 d . . .
O2 O -0.23317(18) 0.85064(13) 0.00816(12) 0.0617(4) Uani 1 1 d . . .
C5 C -0.1493(2) 1.00903(15) 0.19046(14) 0.0383(3) Uani 1 1 d . . .
C9 C -0.0489(2) 1.17530(15) 0.36925(16) 0.0457(4) Uani 1 1 d . . .
H9 H 0.0436 1.2104 0.4378 0.055 Uiso 1 1 calc R . .
C11 C 0.0911(3) 0.67900(18) 0.05557(17) 0.0475(4) Uani 1 1 d . . .
C6 C -0.3272(2) 1.07613(17) 0.16525(16) 0.0481(4) Uani 1 1 d . . .
H6 H -0.4210 1.0429 0.0966 0.058 Uiso 1 1 calc R . .
C8 C -0.2261(3) 1.24057(18) 0.34110(17) 0.0531(4) Uani 1 1 d . . .
H8 H -0.2508 1.3192 0.3909 0.064 Uiso 1 1 calc R . .
C7 C -0.3665(3) 1.19108(18) 0.24037(17) 0.0530(4) Uani 1 1 d . . .
H7 H -0.4864 1.2347 0.2233 0.064 Uiso 1 1 calc R . .

loop_
 _atom_site_aniso_label
 _atom_site_aniso_U_11
 _atom_site_aniso_U_22
 _atom_site_aniso_U_33
 _atom_site_aniso_U_23
 _atom_site_aniso_U_13
 _atom_site_aniso_U_12
C12 0.0466(9) 0.0453(9) 0.0512(10) -0.0115(8) 0.0008(7) 0.0051(7)
C13 0.0478(9) 0.0303(7) 0.0525(9) -0.0052(6) -0.0013(7) 0.0058(6)
C15 0.0577(12) 0.0487(11) 0.0746(14) -0.0050(10) -0.0106(10) 0.0195(9)
C14 0.0774(15) 0.0486(11) 0.0673(13) 0.0035(9) 0.0097(11) -0.0010(10)
O1 0.0491(7) 0.0434(6) 0.0521(7) -0.0189(5) -0.0172(5) 0.0056(5)
N1 0.0408(7) 0.0342(6) 0.0400(7) -0.0083(5) -0.0080(5) 0.0006(5)
O3 0.0449(6) 0.0369(6) 0.0511(6) -0.0096(5) -0.0164(5) 0.0100(4)
C1 0.0335(7) 0.0336(7) 0.0335(7) 0.0006(6) -0.0045(6) -0.0008(6)
C2 0.0326(7) 0.0325(7) 0.0387(8) -0.0009(6) -0.0045(6) 0.0007(6)
C3 0.0366(8) 0.0365(8) 0.0388(8) -0.0060(6) -0.0048(6) -0.0006(6)
C4 0.0371(8) 0.0454(9) 0.0377(8) -0.0033(6) -0.0070(6) -0.0014(6)
C10 0.0374(8) 0.0332(7) 0.0344(7) 0.0011(6) -0.0006(6) 0.0003(6)
O2 0.0519(7) 0.0733(8) 0.0554(7) -0.0193(6) -0.0256(6) 0.0125(6)
```

```
C5 0.0360(8) 0.0411(8) 0.0369(8) 0.0032(6) -0.0026(6) 0.0021(6)
C9 0.0506(9) 0.0391(8) 0.0460(9) -0.0037(7) -0.0062(7) 0.0072(7)
C11 0.0468(9) 0.0476(9) 0.0453(10) -0.0151(7) -0.0067(7) 0.0023(7)
C6 0.0418(9) 0.0534(10) 0.0477(9) -0.0003(7) -0.0093(7) 0.0078(7)
C8 0.0628(11) 0.0459(9) 0.0520(10) -0.0059(7) 0.0011(8) 0.0185(8)
C7 0.0470(10) 0.0554(10) 0.0581(10) 0.0037(8) -0.0008(8) 0.0188(8)

_geom_special_details
;
 All esds (except the esd in the dihedral angle between two l.s. planes)
 are estimated using the full covariance matrix.  The cell esds are taken
 into account individually in the estimation of esds in distances, angles
 and torsion angles; correlations between esds in cell parameters are only
 used when they are defined by crystal symmetry.  An approximate (isotropic)
 treatment of cell esds is used for estimating esds involving l.s. planes.
;

loop_
 _geom_bond_atom_site_label_1
 _geom_bond_atom_site_label_2
 _geom_bond_distance
 _geom_bond_site_symmetry_2
 _geom_bond_publ_flag
C12 C11 1.519(2) . ?
C12 C13 1.525(2) . ?
C12 H12B 0.970(19) . ?
C12 H12A 0.999(18) . ?
C13 O3 1.4732(18) . ?
C13 C15 1.515(2) . ?
C13 C14 1.522(3) . ?
C15 H15A 0.92(3) . ?
C15 H15B 0.96(2) . ?
C15 H15C 1.03(3) . ?
C14 H14A 1.00(2) . ?
C14 H14B 0.99(2) . ?
C14 H14C 0.97(2) . ?
O1 N1 1.3821(15) . ?
O1 H1O 0.85(2) . ?
N1 C1 1.2901(18) . ?
O3 C2 1.3534(17) . ?
C1 C10 1.478(2) . ?
C1 C2 1.482(2) . ?
C2 C3 1.355(2) . ?
C3 C4 1.452(2) . ?
C3 C11 1.511(2) . ?
C4 O2 1.2336(18) . ?
C4 C5 1.492(2) . ?
C10 C9 1.400(2) . ?
C10 C5 1.412(2) . ?
C5 C6 1.392(2) . ?
C9 C8 1.382(2) . ?
C9 H9 0.9300 . ?
C11 H11B 0.996(17) . ?
C11 H11A 0.98(2) . ?
C6 C7 1.379(2) . ?
C6 H6 0.9300 . ?
C8 C7 1.378(2) . ?
```

```
C8 H8 0.9300 . ?
C7 H7 0.9300 . ?

loop_
 _geom_angle_atom_site_label_1
 _geom_angle_atom_site_label_2
 _geom_angle_atom_site_label_3
 _geom_angle
 _geom_angle_site_symmetry_1
 _geom_angle_site_symmetry_3
 _geom_angle_publ_flag
C11 C12 C13 111.59(14) . . ?
C11 C12 H12B 111.1(10) . . ?
C13 C12 H12B 108.6(11) . . ?
C11 C12 H12A 110.4(10) . . ?
C13 C12 H12A 107.3(10) . . ?
H12B C12 H12A 107.8(15) . . ?
O3 C13 C15 103.65(13) . . ?
O3 C13 C14 107.83(14) . . ?
C15 C13 C14 112.00(16) . . ?
O3 C13 C12 108.49(12) . . ?
C15 C13 C12 111.81(15) . . ?
C14 C13 C12 112.53(15) . . ?
C13 C15 H15A 111.6(14) . . ?
C13 C15 H15B 110.4(12) . . ?
H15A C15 H15B 106.6(18) . . ?
C13 C15 H15C 111.2(14) . . ?
H15A C15 H15C 108(2) . . ?
H15B C15 H15C 109.3(18) . . ?
C13 C14 H14A 109.8(13) . . ?
C13 C14 H14B 109.1(13) . . ?
H14A C14 H14B 109.2(18) . . ?
C13 C14 H14C 106.8(13) . . ?
H14A C14 H14C 111.8(18) . . ?
H14B C14 H14C 109.9(18) . . ?
N1 O1 H1O 99.6(15) . . ?
C1 N1 O1 117.23(12) . . ?
C2 O3 C13 118.08(11) . . ?
N1 C1 C10 130.01(13) . . ?
N1 C1 C2 111.74(12) . . ?
C10 C1 C2 118.24(12) . . ?
O3 C2 C3 124.00(13) . . ?
O3 C2 C1 112.97(12) . . ?
C3 C2 C1 123.03(13) . . ?
C2 C3 C4 119.94(14) . . ?
C2 C3 C11 121.33(14) . . ?
C4 C3 C11 118.73(13) . . ?
O2 C4 C3 120.59(14) . . ?
O2 C4 C5 120.76(14) . . ?
C3 C4 C5 118.64(12) . . ?
C9 C10 C5 117.85(14) . . ?
C9 C10 C1 123.94(13) . . ?
C5 C10 C1 118.16(13) . . ?
C6 C5 C10 120.05(14) . . ?
C6 C5 C4 118.50(13) . . ?
C10 C5 C4 121.45(13) . . ?
C8 C9 C10 120.78(15) . . ?
```

```
C8 C9 H9 119.6 . . ?
C10 C9 H9 119.6 . . ?
C3 C11 C12 110.14(13) . . ?
C3 C11 H11B 108.3(10) . . ?
C12 C11 H11B 110.4(10) . . ?
C3 C11 H11A 107.9(11) . . ?
C12 C11 H11A 111.7(11) . . ?
H11B C11 H11A 108.3(14) . . ?
C7 C6 C5 121.03(15) . . ?
C7 C6 H6 119.5 . . ?
C5 C6 H6 119.5 . . ?
C7 C8 C9 121.07(15) . . ?
C7 C8 H8 119.5 . . ?
C9 C8 H8 119.5 . . ?
C8 C7 C6 119.19(15) . . ?
C8 C7 H7 120.4 . . ?
C6 C7 H7 120.4 . . ?

loop_
 _geom_torsion_atom_site_label_1
 _geom_torsion_atom_site_label_2
 _geom_torsion_atom_site_label_3
 _geom_torsion_atom_site_label_4
 _geom_torsion
 _geom_torsion_site_symmetry_1
 _geom_torsion_site_symmetry_2
 _geom_torsion_site_symmetry_3
 _geom_torsion_site_symmetry_4
 _geom_torsion_publ_flag
C11 C12 C13 O3 -59.11(18) . . . . ?
C11 C12 C13 C15 -172.81(14) . . . . ?
C11 C12 C13 C14 60.12(19) . . . . ?
C15 C13 O3 C2 160.86(14) . . . . ?
C14 C13 O3 C2 -80.25(17) . . . . ?
C12 C13 O3 C2 41.89(17) . . . . ?
O1 N1 C1 C10 -1.6(2) . . . . ?
O1 N1 C1 C2 179.34(11) . . . . ?
C13 O3 C2 C3 -11.3(2) . . . . ?
C13 O3 C2 C1 169.91(12) . . . . ?
N1 C1 C2 O3 5.34(17) . . . . ?
C10 C1 C2 O3 -173.80(11) . . . . ?
N1 C1 C2 C3 -173.42(13) . . . . ?
C10 C1 C2 C3 7.4(2) . . . . ?
O3 C2 C3 C4 176.05(13) . . . . ?
C1 C2 C3 C4 -5.3(2) . . . . ?
O3 C2 C3 C11 -3.6(2) . . . . ?
C1 C2 C3 C11 175.00(13) . . . . ?
C2 C3 C4 O2 180.00(15) . . . . ?
C11 C3 C4 O2 -0.3(2) . . . . ?
C2 C3 C4 C5 -1.4(2) . . . . ?
C11 C3 C4 C5 178.24(13) . . . . ?
N1 C1 C10 C9 -4.2(2) . . . . ?
C2 C1 C10 C9 174.80(13) . . . . ?
N1 C1 C10 C5 178.58(14) . . . . ?
C2 C1 C10 C5 -2.46(19) . . . . ?
C9 C10 C5 C6 -2.0(2) . . . . ?
C1 C10 C5 C6 175.42(13) . . . . ?
```

```
C9 C10 C5 C4 178.53(13) . . . . ?
C1 C10 C5 C4 -4.0(2) . . . . ?
O2 C4 C5 C6 5.3(2) . . . . ?
C3 C4 C5 C6 -173.24(13) . . . . ?
O2 C4 C5 C10 -175.20(14) . . . . ?
C3 C4 C5 C10 6.2(2) . . . . ?
C5 C10 C9 C8 1.4(2) . . . . ?
C1 C10 C9 C8 -175.85(14) . . . . ?
C2 C3 C11 C12 -14.3(2) . . . . ?
C4 C3 C11 C12 166.00(14) . . . . ?
C13 C12 C11 C3 45.3(2) . . . . ?
C10 C5 C6 C7 1.0(2) . . . . ?
C4 C5 C6 C7 -179.49(14) . . . . ?
C10 C9 C8 C7 0.2(3) . . . . ?
C9 C8 C7 C6 -1.2(3) . . . . ?
C5 C6 C7 C8 0.6(3) . . . . ?

_diffrn_measured_fraction_theta_max    0.995
_diffrn_reflns_theta_full              25.00
_diffrn_measured_fraction_theta_full   0.995
_refine_diff_density_max    0.137
_refine_diff_density_min   -0.190
_refine_diff_density_rms    0.041
```
